# The impact of COVID-19 on cancer care in a tertiary hospital in Korea: possible collateral damage to emergency care

**DOI:** 10.4178/epih.e2022044

**Published:** 2022-05-01

**Authors:** Shin Hye Yoo, Jin-Ah Sim, Jeongmi Shin, Bhumsuk Keam, Jun-Bean Park, Aesun Shin

**Affiliations:** 1 Center for Palliative Care and Clinical Ethics, Seoul National University Hospital, Seoul, Korea; 2 School of AI Convergence, Hallym University, Chuncheon, Korea; 3 Department of Internal Medicine, Seoul National University Hospital, Seoul, Korea; 4 Department of Preventive Medicine, Seoul National University College of Medicine, Seoul, Korea; 5 Cancer Research Institute, Seoul National University, Seoul, Korea

**Keywords:** COVID-19, Cancer, Care, Tertiary hospital, Republic of Korea

## Abstract

**OBJECTIVES:**

We investigated the impact of the COVID-19 pandemic on cancer care in a tertiary hospital in Korea without specific lockdown measures.

**METHODS:**

A retrospective cohort of cancer patients from one of the largest tertiary hospitals in Korea was used to compare healthcare utilization in different settings (outpatient cancer clinic, the emergency department [ED], and admissions to the hematology/oncology ward) between January 1 and December 31, 2020 and the same time period in 2019. The percent changes in healthcare utilization between the 2 periods were calculated.

**RESULTS:**

A total of 448,833 cases from the outpatient cohort, 26,781 cases from the ED cohort, and 14,513 cases from the admission cohort were reviewed for 2019 and 2020. The total number of ED visit cases significantly decreased from 2019 to 2020 by 18.04%, whereas the proportion of cancer patients remained stable. The reduction in ED visits was more prominent in patients with symptoms suspicious for COVID-19, high-acuity cases, and those who lived in non-capital city areas. There were no significant changes in the number of total visits, new cases in the outpatient clinic, or the total number of hospitalizations between the 2 periods.

**CONCLUSIONS:**

During the pandemic, the number of ED visits significantly decreased, while the use of the outpatient clinic and hospitalizations were not affected. Cancer patients’ ED visits decreased after the COVID-19 outbreak, suggesting the potential for collateral damage outside the hospital if patients cannot reach the ED in a timely manner.

## INTRODUCTION

Since coronavirus disease 2019 (COVID-19) was declared a pandemic in March 2020 by the World Health Organization [1], 2.55 billion cases and 5.12 million deaths have been reported worldwide [2]. This emerging new virus, severe acute respiratory syndrome coronavirus 2 (SARS-CoV-2), threatened the overall healthcare system and disrupted healthcare delivery [3].

Cancer patients have been widely affected by the spread of COVID-19 because they are more vulnerable to SARS-CoV-2 infection [4] and more likely to have negative outcomes than patients without cancer [5,6]. A critical issue in cancer care is the adequate prevention, diagnosis, treatment, and management of those affected in a timely manner [7], and failure to carry out any of these procedures is of concern. In particular, the continuum of cancer care can be severely affected by limitations in healthcare resources resulting from pandemic-related lockdowns [8]. The consensus statements from oncological societies such as the American Society of Clinical Oncology and the European Society of Medical Oncology suggest that the risk of exposure to infection should be minimized by applying proper precautionary measures, and that patient care should be prioritized based on the individual condition and the status of healthcare in each region. It is also emphasized that curative interventions for cancer should not be delayed if possible [9,10].

Korea is no exception to the COVID-19 pandemic, and the nation has been affected by COVID-19 during 3 main waves of infection. The first confirmed COVID-19 case was reported on January 20, 2020, and 60,740 cases were confirmed as of December 31, 2020, with 900 COVID-19-related deaths occurring during this period. Korea has implemented social distancing, as well as extensive testing and tracing, and face masks are worn in public; persons with COVID-19 undergo quarantine, although there are no national lockdown measures [11].

COVID-19 disrupted cancer care during the lockdown period in countries such as the United States [8], the Netherlands [12], and India [13], whereas in Korea, even the early first wave of COVID-19 did not affect cancer care significantly throughout the country [14,15]. However, healthcare utilization by cancer patients in Korea has not been investigated in detail. In this study, we investigated the impact of COVID-19 on healthcare utilization in various clinical settings in a tertiary hospital in Korea.

## MATERIALS AND METHODS

### Study design and study participants

To estimate the impact of the COVID-19 pandemic on healthcare utilization in a tertiary hospital in Korea, we collected data on outpatient visits, emergency care, and the admissions registry of Seoul National University Hospital (SNUH). SNUH is one of the largest tertiary hospitals in Korea; it is a 1,761-bed tertiary referral hospital that employs 1,947 doctors for acute and specialized care. Although the hospital is located in the central capital city area, not all patients are from the capital, and the hospital treats patients from all over the country. In addition, the emergency department (ED) of SNUH has been a regional emergency center since 2000.

Patients older than 19 years of age and patients diagnosed with International Classification of Diseases, 10th revision (ICD-10) codes of C00-C90 or D45-D48 were selected from the electronic medical record (EMR) system, and clinical data were retrieved from the SNUH Patient Research Environment platform. Patients who met the ICD-10 code criterion and who visited the outpatient clinic at least 3 times or visited the ED or were hospitalized at least once were included in the analysis. Data were obtained from the following 3 retrospective cohorts: (1) an outpatient cohort, (2) an ED cohort, and (3) an inpatient cohort. The outpatient cohort included patients who visited the outpatient cancer center of SNUH. The ED cohort included patients who visited the ED of SNUH. The inpatient cohort included patients who were hospitalized in the hematology-medical oncology department of SNUH. Patients with missing information about their cancer diagnosis and treatment in the EMR were excluded. Data were collected from the 3 cohorts between January 1, 2019 and December 31, 2020. The period between January 1, and December 31, 2020 was designated as the during-COVID-19 period. This period included 3 COVID-19 outbreaks (first wave: February 20 to March 9, 2020, primarily in Daegu; second wave: May 6 to August 10, 2020, primarily in the capital area; and third wave: November 2020 to January 2021, national spread). The same time periods during 2019 were defined as the pre-COVID-19 period to compare patients before and after COVID-19.

### Data collection and measurements

The data obtained included demographics such as age, sex, identification number, address, type of medical insurance, and cancer type. Patients were divided into 12 groups according to cancer type based on the ICD-10 code classification as follows: lip, oral cavity, and pharynx (C00-C14); gastrointestinal (C15-C21, C26); hepatobiliary-pancreas (C22-C25); lung and intrathoracic (C30-C39); bone and soft tissue (C40-C41, C45-C49); breast (C50); gynecological (C51-C58); genitourinary (C60-C68); lymphoma (C81-C85); leukemia (C91-C95); other hematological (C86, C88, C90, C96, D45-D48); and others. Others included malignant neoplasms of the skin (C43-C44), central nervous system (C69-C72), endocrine glands (C73-C75), and metastatic or of unknown origin (C76-C80). In the outpatient cohort, the numbers of outpatient visits and new cases were collected. For patients scheduled to be admitted, the delay time of the planned admission was calculated as the time between the actual admission date and the expected admission date. For the ED cohort, data collected included the total number of visits, the chief complaints, the reason for the ED visit (disease or non-disease cause), the route of the ED visit (referred from an outpatient clinic, from home, or via another hospital), the length of ED stays, the discharge outcomes (home, transfer, admission, or death), and the patients’ acuity level at triage as assessed by the Korean Triage and Acuity Scale (KTAS). The KTAS was developed in 2012 [16]. To classify the KTAS level, well-trained ED staff assess the critical first-look of the patient and investigate the patient’s symptoms, with primary (characteristics common to most symptoms and signs such as consciousness, blood pressure, heart rate, respiration rate, fever, pain, presence of hemorrhage, and trauma) and secondary factors (characteristics applied to specific symptoms). This triage scale has 5 categories ranging from KTAS 1, which requires immediate aggressive treatment due to life-threatening conditions, to KTAS 5, which is a non-emergency visit due to chronic illness [17]. In general, patients with KTAS 1-3 are considered emergency patients, and those with KTAS 4 and 5 are considered non-emergency patients.

If the chief complaints were fever ≥ 37.5°C or respiratory symptoms such as coughing or difficulty breathing, patients were classified as having symptoms suspicious for COVID-19. Other chief complaints were classified as “others.” Data collected for the inpatient cohort included the number of admissions, the length of hospital stay, discharge outcome (death or non-death), and route of admission (via an outpatient clinic or via the ED). In addition, for the inpatient cohort, the fatality rate and the proportion of admissions through the ED were calculated as follows:

• $\;\mathrm{Fatality}\;\mathrm{rate}\;(\%)=\frac{\mathrm{the}\;\mathrm{number}\;\mathrm{of}\;\mathrm{deaths}}{\mathrm{all}\;\mathrm{admissions}}\times 100$

• $\mathrm{The}\;\mathrm{proportion}\;\mathrm{of}\;\mathrm{admissions}\;\mathrm{through}\;\mathrm{the}\;\mathrm{ED}=\frac{\mathrm{admissions}\;\mathrm{through}\;\mathrm{the}\;\mathrm{ED}}{\mathrm{all}\;\mathrm{admissions}}$

### Statistical analysis

This retrospective study assessed whether changes in the utilization of different health services (outpatient clinic visits, ED visits, and admissions) were affected by the COVID-19 pandemic. Baseline demographics collected from the 2019 and 2020 registries were analyzed using descriptive statistics (means and median values, frequencies, and percentages) using the Student t-test and the chi-square test. The p-values (α) < 0.05 were considered to indicate statistical significance. For each service category, the number of cases registered in the hospital’s EMR was tracked, trends were evaluated, and the mean percent change in admission delays (2019 vs. 2020) was estimated and compared. In addition, a sensitivity analysis was conducted by stratifying the number of events that occurred monthly during the calendar period 2019-2020 by age, sex, region, and cancer type, and the results were plotted. In some instances, the relative percent changes (decrease or increase) of events occurring in 2020 versus the same period in 2019 were presented. Statistical analyses were performed using SAS version 9.4 (SAS Institute Inc., Cary, NC, USA), and graphs were plotted using Microsoft Excel version 2016 (Microsoft Corp., Redmond, WA, USA).

### Ethics statement

The study protocol was approved by the Institutional Review Board of SNUH (IRB No. H-2104-102-1212). The requirement for informed consent was waived.

## RESULTS

### Demographic characteristics of study cases

A total of 222,307 cases and 226,526 cases visited the outpatient clinic in 2019 and 2020, respectively (note that 1 patient can have redundant cases due to multiple visits). The number of patients who visited the outpatient clinic was 74,547 in 2019 and 75,026 in 2020. In 2020, 7,151 patients (12,288 cases) visited the ED, which was lower than the 8,347 patients (14,993 cases) in 2019. The numbers of hospitalized patients and cases in 2020 (2,741 patients and 7,397 cases) did not differ significantly from those in 2019 (2,774 patients and 7,116 cases). The differences in demographic and clinical characteristics of outpatient clinic visits, ED visits, and admission cases between 2019 and 2020 are described in Table 1. There were significant differences in age, insurance, and residence among outpatients and ED patients between 2019 and 2020 (p<0.05 for all), whereas there were no marked differences in sex and residence in the admission cohort. Regarding cancer type, outpatient clinic visits for gastrointestinal cancers (p=0.001) and lymphoma (p=0.04) differed significantly between 2019 and 2020.

### Outpatient clinic visits

**Figure 1.**
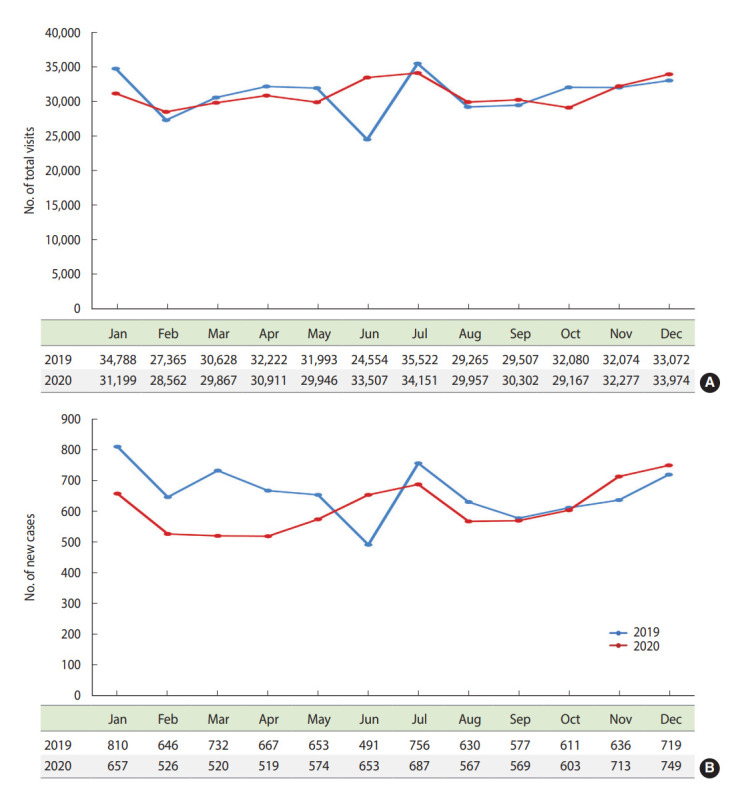
Number of outpatient clinic visits during the pre-COVID-19 and COVID-19 periods. (A) Total number of outpatient clinic visits. (B) Number of new cases in the outpatient clinic. COVID-19, coronavirus disease 2019.

**Figure 2.**
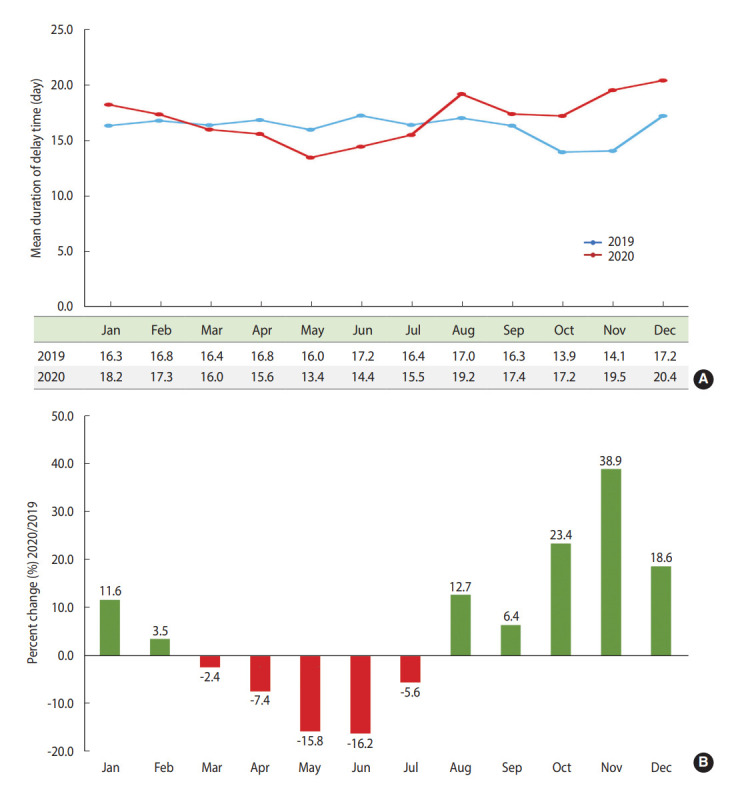
Delay time for planned admissions during the pre-COVID-19 and COVID-19 periods. (A) Average delay time for planned admissions. (B) Percent changes of delay time for planned admissions. COVID-19, coronavirus disease 2019.

The total number of outpatient clinic visits was compared between 2019 and 2020 (Figure 1A). There were no marked differences between the 2 years, although the number of patients who visited the outpatient clinic for the first time showed a continuously decreasing trend during the first quarter of 2020 (657→526→520) compared with the same period in 2019 (810→646→732) (Figure 1B). Subgroup analyses of these visits did not show marked differences according to age, sex, insurance, residence, and cancer diagnosis (Supplementary Material 1). Delays in admission were investigated in 28,148 cases with planned admission orders from the outpatient clinic (Supplementary Material 2), and data on the admission delays are shown in Figure 2. After the second and third COVID-19 waves in 2020 (which occurred nationwide), admission delays increased by 12.7% in August 2020 and by 23.4-36.9% during October and November of 2020 compared with the same period of 2019. Subgroup analyses showed slight differences in the percent changes of admission delays between 2019 and 2020 according to age, sex, and insurance (Supplementary Material 3).

### Emergency department visits

**Figure 3.**
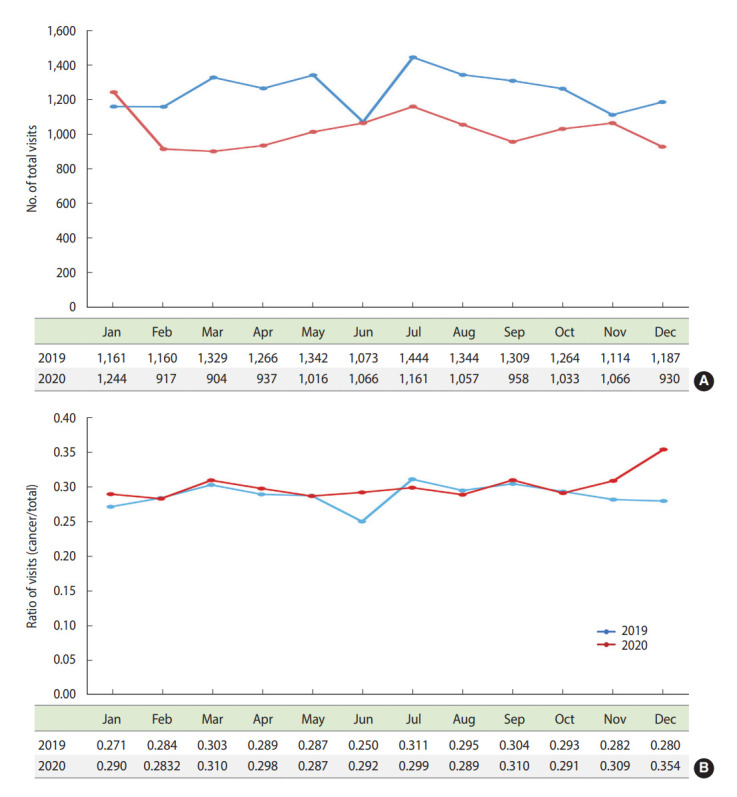
ED visits during the pre-COVID-19 and COVID-19 periods. (A) Total number of ED visits. (B) The ratio of ED visits by cancer patients to that of all patients who visited the ED. ED, emergency department; COVID-19, coronavirus disease 2019.

**Figure 4.**
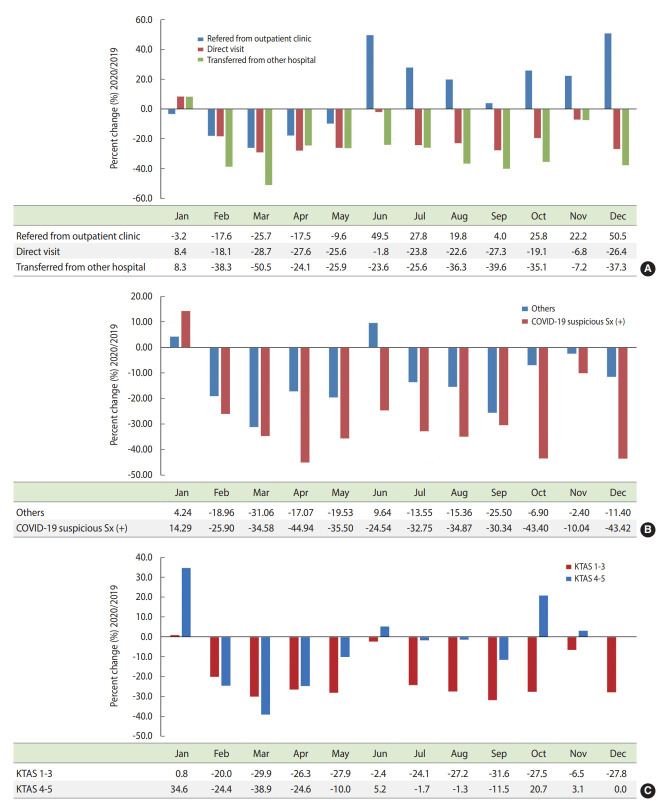
Percent changes in monthly ED visits stratified by various factors. (A) Percent changes of ED visits by route of visit. (B) Percent changes of ED visits by chief complaint. (C) Percent changes of ED visits by KTAS level. ED, emergency department; KTAS, Korean Triage and Acuity Scale; Sx, symptoms.

Overall, the number of total ED visits decreased in 2020 (from 1,244 cases in January to 930 cases in December) compared with the same period in 2019 (from 1,161 cases in January to 1,187 cases in December) (Figure 3A). However, the ratio of ED visits of cancer patients to all patients who visited the ED was higher in December 2020 (0.354) than in the same period of 2019 (0.280) (Figure 3B). The percent changes in the monthly route of ED visits stratified by various clinical factors were also investigated (Figure 4). The percentage of patients referred from the outpatient clinic increased in the second half of 2020 (range, 4.0 to 50.5%), whereas the percentage of patients transferred from another hospital (range, -50.5 to -7.2%) or who visited by themselves (range, -28.7 to -1.8%) decreased during 2020 (Figure 4A). The percent changes of monthly ED visits according to the chief complaint also differed between 2019 and 2020. When patients who visited the ED showed symptoms suspicious for COVID-19, the number of ED visits decreased even more (range, -44.9 to -10.0%) than in other cases (range, -19.0 to 2.4%) (Figure 4B). The changes in monthly ED visits between 2019 and 2020 according to KTAS level were also investigated. ED visits decreased more markedly in KTAS levels 1-3 than in KTAS levels 4 and 5 during 2020 (p < 0.001) (Figure 4C). The percent changes of monthly ED visits by age and sex varied slightly from month to month (Supplementary Material 4). For the third wave, the percent changes of monthly ED visits (2019/2020) decreased more markedly for residents in non-capital city areas than in the capital city area (Supplementary Material 4). Visits for disease-related causes decreased, whereas visits for nondisease causes remained the same (Supplementary Material 4). The numbers of monthly ED visits by cancer diagnosis are described in Supplementary Material 5.

The length of ED stays significantly decreased from 2019 to 2020 (mean±standard deviation: 11.38±13.47 vs. 8.58±9.88 hours, respectively; p < 0.001). The proportion of patients discharged directly home from the ED decreased, and the percentage of patients transferred to another hospital also decreased. However, the number of deaths in the emergency room increased in 2020 (Supplementary Material 6). In the sub-analysis according to the presence of symptoms suspicious for COVID-19, the monthly ED discharge results differed between 2019 and 2020 (Supplementary Material 6).

### Admissions

In the inpatient cohort, there were no significant differences in the number of admissions and the average length of hospital stay between 2019 and 2020 (Supplementary Material 7). There were no major differences in the subgroup analysis by age, sex, insurance, and region (Supplementary Material 8). Because the ED visits in the ED cohort differed between 2019 and 2020, we additionally analyzed the proportion of admissions through the ED in 2019 and 2020. Although the overall proportion of admissions through the ED did not differ (Supplementary Material 7), that of residents of the capital city area was lower in 2020 than in 2019 (Supplementary Material 7). The average fatality rate of the inpatient cohort did not differ between 2019 and 2020 (n=267, 3.6% in 2019 vs. n=224, 3.1% in 2020; p=0.124).

## DISCUSSION

The COVID-19 pandemic challenged proper and timely cancer care because of treatment delays and limited access to healthcare systems in several countries. In this study, we showed that the COVID-19 pandemic caused an 18.04% decrease in ED visits by cancer patients, especially in patients with symptoms suspicious for COVID-19, patients who directly visited the ED or were transferred from other hospitals, and patients with a higher degree of acuity. Although the volume of outpatient clinic visits and inpatient hospitalizations, which mainly corresponded to planned care, remained stable, the emergency care of cancer patients was altered during the COVID-19 pandemic.

In this study, the total number of outpatient clinic visits remained stable during-the COVID-19 period compared with that in the pre-COVID-19 period, and a similar pattern was observed in the number of admissions in the inpatient cohort. In addition, the number of new outpatient clinic patients surged after a slight decline during the first wave of infection. This is consistent with a study conducted in a large tertiary hospital in Korea, which showed that cancer diagnosis, treatment, and admissions decreased slightly immediately after the first COVID-19 wave [14]. However, the decrease in outpatient clinic visits was more prominent in other countries [8,13,18,19], especially in those that adopted lockdown measures [20]. Although the Daegu and Gyeongbuk areas were significantly affected by the first wave of the COVID-19 pandemic, which led to collateral damage in Korea [21,22], these local impacts may not have spread to outpatient clinics or inpatient hospitalizations in other distant areas. Korea maintained national strategies for COVID-19 control without adopting complete lockdown measures. According to the national policy, which included early recognition and response, developing a prompt and accurate diagnostic capacity, and strengthening community-level preventive measures [23], SNUH implemented the following strategies to protect patients and healthcare providers: establishing an infectious disease crisis response committee; restricting access to the hospital, as well as fever and respiratory symptom screening for all visitors; operating separate respiratory clinics to guarantee public safety; operating a COVID-19 screening center in the hospital; designation of a pre-emptive isolation room for patients with suspicious symptoms; and restricting visitor access for high-risk units such as the intensive care unit. These measures allowed SNUH to optimize hospital capacity for cancer care without restricting cancer center visits or hospitalizations.

Nonetheless, the decrease in ED visits of cancer patients found in the present study indicates that emergency or urgent care for cancer patients may not have been adequately provided. We also observed that the decrease in ED visits was more prominent after the second wave, in which the outbreak occurred primarily in the capital city area. Providing adequate, acute care in the ED is critical for cancer patients, who may experience acute symptoms and signs resulting from the cancer itself or as complications of anticancer treatments [24,25]. During a pandemic, the ED is one of the most vulnerable hospital departments because of the increased volume of emergency visits and disruptions of the previous healthcare delivery system [26-28]. The regional ED is a particularly valuable resource [29,30], and in addition to cancer patients, those with serious medical problems rather than those with mild symptoms should visit the ED. However, in this study, we observed the opposite pattern, suggesting that patients with higher acuity or those with fever or respiratory symptoms did not visit the ED.

Although the reasons for the decreased ED visits were not investigated in this study, we suggest 3 hypothetical scenarios. First, it is possible that the incidence of fever or respiratory symptoms in cancer patients as a potential chief complaint for ED visits remained unchanged, but some patients were systematically blocked from ED utilization. In Korea, patients visiting the ED directly or being transferred from other hospitals who present with symptoms and/or signs suspicious for COVID-19 are first placed in an isolated room in the ED until a COVID-19 test result is obtained. If the ED does not have a vacant isolation room, it cannot accommodate the patient, regardless of the severity of the problem. This hypothesis is supported by a report of changes in the emergency medical system during the pandemic from Gyeonggi-do in Korea showing that the length of transfer time to the hospital by 119 ambulances increased more in febrile patients than non-febrile patients [31]. Similarly, at another tertiary hospital in Korea, the rates of leaving without being seen significantly increased in patients with fever or respiratory symptoms in comparison to those without the symptoms [32], which may reflect a failure of the ED process [33]. In addition, as the ED bed capacity is limited, the different routes of ED visits are usually mutually exclusive. The findings of this study that direct ED visits or transfers from other hospitals decreased, whereas transfers from the outpatient clinic of the same hospital remained constant, may support the possibility that the emergency medical system applied to severe patients bypasses the ED when the ED is full. This is consistent with a previous report showing that transfer-in visits decreased during the pandemic because of scarce isolation rooms [31]. This suggests that cancer patients experienced longer wait times for emergency care by detouring the ED and they might have missed timely management. Second, patients may avoid the ED because of various factors. For example, cancer patients and their caregivers may be afraid of being infected with COVID-19 or exposed to anonymous COVID-19 patients in a crowded ED [34-36]. Third, the actual incidence of respiratory infectious diseases in cancer patients may have been reduced because of a high level of social distancing and behavioral changes (e.g., maintaining good personal hygiene such as wearing a mask and regularly washing one’s hands). This hypothesis is supported by a Korean national retrospective study showing that a reduction in the diagnosis of respiratory infectious diseases was observed among febrile patients who visited the ED in 2020 compared with 2019 [37].

Although the underlying reason remains unclear, we found that the reduction of ED visits by more severe patients (KTAS levels 1-3) was more prominent than the reduction in visits by less severe patients (KTAS levels 4 and 5), along with the overall decrease in ED visits. Similar phenomena were observed in other studies [38,39]. Patients classified as KTAS level 1 (resuscitation) and KTAS level 2 (emergent) usually have unstable vital signs and come to the ED by ambulance (119 and non-119). In addition, most of symptoms assessed as a high KTAS level include fever or respiratory symptoms such as dyspnea, or decreased mentality, which need an isolation room when approaching the ED. Considering these reasons and the potential blockade of the ED caused by insufficient resources, more severe patients may be more likely not to enter the ED than less severe patients.

The length of ED stays increased, and the number of deaths in the ED increased during the pandemic. Similarly, another study of all patients, including cancer and non-cancer patients, also showed that fatalities in the ED increased during the COVID-19 period [31]. Despite the lower severity of the patients who visited the ED in 2020 than in 2019, the length of ED stays increased due to several problems such as precautionary procedures for COVID-19 prevention or difficulties in transfers to other hospitals for patients who could not be accommodated in SNUH [31]. Regardless of the reason, prolonged ED stays may be associated with worsened medical conditions of patients who need hospitalization and proper care, leading to deaths in the ED [40].

In this study, the marked decrease of ED visits by residents of the non-capital city areas compared to residents of the capital city area suggests the presence of differences in ED accessibility according to patients’ residence. Cancer-related ED visits are known to be associated with visits to certificated tertiary hospitals, metropolitan regions, and a higher KTAS level (such as emergent or urgent) [41]. As SNUH is located in the center of the capital city, patients who live in non-capital city areas may visit the ED for cancer-related acute problems at local hospitals, not SNUH, due to the physical distance. The difficulty in transfer from other hospitals to SNUH during the pandemic could explain the difference in the reduction of ED visits according to the area of residence.

This study had several limitations. First, the study was conducted at a single center (in a tertiary hospital in Korea), and the findings cannot be generalized to all cancer patients in other hospitals. Second, we examined the number of visits and admissions rather than the incidence or prevalence of cancer diagnosis. The number of visits or admissions may not reflect the annual volume of cancer diagnoses or the volume of patients receiving specific anticancer treatments such as surgery, chemotherapy, or radiotherapy. Third, the retrospective nature of this study, which was based on the review of medical records, prevented the comprehensive acquisition of data needed to explain the changes in visits. There may have been unrecognized factors that affected the study findings. However, it was difficult to construct a prospective cohort of overall cancer patients during the pandemic.

In conclusion, the results of this retrospective cohort study indicate that cancer patients’ visits to the ED decreased after the outbreak of COVID-19, and suggest the potential for collateral damage outside the hospital for patients unable to utilize the emergency room in time. The most vulnerable place in cancer care management during the pandemic period was the emergency room, and use of the ED directly affects treatment decisions by cancer patients. Given that the COVID-19 pandemic is ongoing in Korea, relevant data are important for policy development to ensure efficient ED use, which is essential to improve the quality of cancer care, especially in the context of limited medical resources. During this pandemic era, patients should feel confident about utilizing the ED and receiving safe and appropriate cancer care without any delays, and if further investigations are required, patients should be referred promptly to clinicians to reduce excess deaths.

## Figures and Tables

**Table 1.** Baseline characteristics of cancer patients from the outpatient cancer center who visited the emergency department and were admitted to the hematology/oncology ward

Characteristics		Outpatient clinic			Emergency department			Admission		
		2019 (n=74,547)	2020 (n=75,026)	p-value	2019 (n=8,347)	2020 (n=7,151)	p-value	2019 (n=2,774)	2020 (n=2,741)	p-value
Age										
	Median (Q1-Q3)	60.55 (51.35-69.30)	61.01 (51.59-69.66)	<0.001	64.39 (55.70-72.88)	63.04 (53.78-72.01)	<0.001	62.52 (53.56-70.29)	61.87 (52.24-69.15)	<0.001
Sex										
	Male	41,975 (56.3)	42,468 (56.6)	0.246	3,896 (46.7)	3,286 (45.9)	0.368	1,555 (56.1)	1,495 (54.5)	0.258
	Female	32,572 (43.7)	32,558 (43.4)		4,451 (53.3)	3,865 (54.0)		1,219 (43.9)	1,246 (45.5)	
Insurance										
	National insurance	72,105 (96.7)	72,637 (96.8)	<0.001	7,913 (94.8)	6,769 (94.7)	0.010	2,647 (95.4)	2,630 (95.9)	0.028
	Medical Aid	2,100 (2.8)	2,208 (2.9)		409 (4.9)	376 (5.2)		114 (4.1)	108 (3.9)	
	Others	342 (0.5)	181 (0.2)		24 (0.3)	6 (0.1)		14 (0.5)	3 (0.1)	
Residence										
	Capital city area^1^	52,665 (70.6)	53,508 (71.3)	0.004	6,709 (80.4)	5,916 (82.7)	<0.001	1,862 (67.1)	1,850 (67.5)	0.769
	Non-capital city area	21,877 (29.3)	21,516 (28.7)		1,638 (19.6)	1,235 (17.3)		912 (32.9)	891 (32.5)	
Cancer type^2^										
	Lip, oral cavity, and pharynx	1,085 (1.4)	1,094 (1.5)	0.966	120 (1.4)	101 (1.4)	0.893	85 (3.1)	87 (3.2)	0.818
	Gastrointestinal	10,402 (18.6)	13,581 (17.9)	0.001	1,467 (17.5)	1,250 (17.4)	0.915	494 (17.8)	502 (18.3)	0.634
	Hepatobiliary-pancreas	8,015 (10.7)	7,975 (10.6)	0.462	1,996 (23.9)	1,796 (25.1)	0.077	369 (13.3)	352 (12.8)	0.601
	Lung and intrathoracic	7,780 (10.3)	7,850 (10.4)	0.865	1,155 (13.8)	915 (12.8)	0.056	443 (15.9)	419 (15.2)	0.477
	Bone and soft tissue	73,212 (98.0)	73,721 (98.1)	0.473	188 (2.2)	161 (2.2)	0.999	161 (5.8)	160 (5.8)	0.963
	Breast	15,699 (20.9)	15,921 (21.1)	0.428	904 (10.8)	716 (10.0)	0.099	201 (7.2)	237 (8.6)	0.056
	Gynecological	1,950 (2.6)	2,082 (2.8)	0.058	439 (5.3)	381 (5.3)	0.848	11 (0.4)	11 (0.4)	0.979
	Genitourinary	8,051 (10.7)	8,298 (11.0)	0.107	694 (8.3)	648 (9.0)	0.098	80 (2.9)	92 (3.3)	0.315
	Lymphoma	1,680 (2.2)	1,575 (2.1)	0.040	218 (2.6)	165 (2.3)	0.227	409 (14.7)	376 (13.7)	0.271
	Leukemia	949 (1.3)	1,000 (1.3)	0.309	182 (2.2)	185 (2.6)	0.098	186 (6.7)	187 (6.8)	0.868
	Other hematological	1,090 (1.5)	1,152 (1.5)	0.244	395 (4.7)	334 (4.7)	0.850	213 (7.7)	211 (7.7)	0.985
	Others	13,013 (17.3)	13,117 (17.3)	0.822	513 (6.1)	424 (5.9)	0.561	126 (4.5)	113 (4.1)	0.441

Values are presented as number (%). ICD-10, International Classification of Diseases, 10th revision.

1 The capital city area includes Seoul, Gyeonggi Province, and Incheon.

2 Cancer type classification (by ICD-10 codes): lip, oral cavity, and pharynx (C00-C14); gastrointestinal (C15-C21, C26); hepatobiliary-pancreas (C22-C25); lung and intrathoracic (C30-C39); bone and soft tissue (C40-C41, C45-C49); breast (C50); gynecological (C51-C58); genitourinary (C60-C68); lymphoma (C81-C85); leukemia (C91-C95); other hematological (C86, C88, C90, C96, D45-D48); others including malignant neoplasms of the skin (C43-44), central nervous system (C69-C72), endocrine glands (C73-C75), and metastatic or unknown origin (C76-C80).

## SUPPLEMENTARY MATERIALS

Supplementary Material 1. Monthly outpatient visits by demographic characteristics

Supplementary Material 2. Flow chart of the analysis of the delay time for planned admission. (All cases were investigated from January 2019 to December 2020)

Supplementary Material 3. Percent change of delay time for planned admission according to various factors. (A) Percent change of delay time for planned admission by age group. (B) Percent change of delay time for planned admission by sex. (C) Percent change of delay time for planned admission by type of insurance.

Supplementary Material 4. Percent changes of monthly ED visits stratified by various factors. (A) Percent changes of monthly ED visits by age group. (B) Percent change of monthly ED visits by sex. (C) Percent change of ED visits by residence. (D) Percent change of monthly ED visits by reason for visit. ED, emergency department.

Supplementary Material 5. Number of monthly ED visits by cancer type

Supplementary Material 6. Discharge from emergency department outcomes for the pre-COVID-19 and during-COVID-19 periods and their associations with chief complaints. (A) Monthly discharge outcome from the emergency department. (B) Discharge to home as the outcome stratified by chief complaint. (C) Transfer to other hospital as discharge outcome stratified by chief complaint. (D) Admission as discharge outcome stratified by chief complaint.

Supplementary Material 7. Hospital admissions in the pre-COVID-19 and during-COVID-19 periods. (A) Number of admissions. (B) Average length of hospital stay. (C) Overall rate of admissions via the emergency department. (D) Rate of admissions via the emergency department stratified by residence.

Supplementary Material 8. Monthly cases of admission stratified by demographic characteristics
