# Supplementary material for: The impact of COVID-19 on cancer care in a tertiary hospital in Korea: possible collateral damage to emergency care
Source: Epidemiol Health. 2022 May 1;44:e2022044. doi: 10.4178/epih.e2022044 (PMC9684015; doi:10.4178/epih.e2022044)
Supplement: Supplementary Material 1. — Monthly outpatient visits by demographic characteristics [file epih-44-e2022044-suppl1.docx]

**Supplementary Material 1. Monthly outpatient visits by demographic characteristics**

| Variables | Description | Year | Jan | Feb | Mar | Apr | May | Jun | Jul | Aug | Sep | Oct | Nov | Dec |
| --- | --- | --- | --- | --- | --- | --- | --- | --- | --- | --- | --- | --- | --- | --- |
| **Sex** | Female | 2019 | 443 | 337 | 374 | 323 | 318 | 245 | 385 | 330 | 278 | 301 | 311 | 393 |
|  |  | 2020 | 350 | 279 | 256 | 276 | 301 | 348 | 371 | 285 | 273 | 309 | 371 | 395 |
|  | Male | 2019 | 367 | 309 | 358 | 344 | 335 | 246 | 371 | 300 | 299 | 310 | 325 | 326 |
|  |  | 2020 | 307 | 247 | 264 | 243 | 273 | 305 | 316 | 282 | 296 | 294 | 342 | 354 |
| **Age group (years)** | 20–30s | 2019 | 73 | 55 | 70 | 68 | 49 | 41 | 85 | 56 | 61 | 70 | 58 | 66 |
|  |  | 2020 | 61 | 46 | 48 | 62 | 62 | 73 | 63 | 48 | 59 | 62 | 66 | 83 |
|  | 40–50s | 2019 | 383 | 279 | 331 | 286 | 322 | 215 | 320 | 288 | 231 | 253 | 287 | 320 |
|  |  | 2020 | 304 | 233 | 225 | 220 | 233 | 257 | 288 | 251 | 232 | 249 | 305 | 345 |
|  | 60–70s | 2019 | 328 | 295 | 300 | 290 | 262 | 225 | 323 | 263 | 265 | 273 | 272 | 309 |
|  |  | 2020 | 272 | 230 | 235 | 221 | 256 | 293 | 312 | 250 | 253 | 270 | 315 | 293 |
|  | ≥80s | 2019 | 26 | 17 | 31 | 23 | 20 | 10 | 28 | 23 | 20 | 15 | 19 | 24 |
|  |  | 2020 | 20 | 17 | 12 | 16 | 23 | 30 | 24 | 18 | 25 | 22 | 27 | 28 |
| **Insurance** | National Health Insurance | 2019 | 790 | 613 | 701 | 629 | 616 | 465 | 730 | 602 | 550 | 589 | 600 | 693 |
|  |  | 2020 | 632 | 502 | 502 | 498 | 554 | 631 | 657 | 549 | 543 | 573 | 690 | 729 |
|  | Medicaid | 2019 | 16 | 25 | 21 | 27 | 29 | 19 | 19 | 21 | 18 | 13 | 25 | 20 |
|  |  | 2020 | 16 | 16 | 15 | 20 | 16 | 20 | 27 | 15 | 23 | 22 | 18 | 17 |
|  | Others | 2019 | 4 | 8 | 10 | 11 | 8 | 7 | 7 | 7 | 9 | 9 | 11 | 6 |
|  |  | 2020 | 9 | 8 | 3 | 1 | 4 | 2 | 3 | 3 | 3 | 8 | 5 | 3 |
| **Residence** | Capital city area^a)^ | 2019 | 490 | 385 | 438 | 382 | 405 | 307 | 451 | 366 | 354 | 357 | 364 | 453 |
|  |  | 2020 | 385 | 347 | 311 | 282 | 351 | 391 | 431 | 349 | 340 | 352 | 419 | 464 |
|  | Non-capital city area | 2019 | 320 | 261 | 294 | 284 | 248 | 184 | 305 | 264 | 223 | 253 | 271 | 266 |
|  |  | 2020 | 272 | 179 | 209 | 237 | 223 | 262 | 256 | 218 | 229 | 250 | 294 | 285 |

^a)^ Capital city area includes Seoul-si, Gyeonggi-province, and Incheon-si.
