# Supplementary material for: The impact of COVID-19 on cancer care in a tertiary hospital in Korea: possible collateral damage to emergency care
Source: Epidemiol Health. 2022 May 1;44:e2022044. doi: 10.4178/epih.e2022044 (PMC9684015; doi:10.4178/epih.e2022044)
Supplement: Supplementary Material 2. — Flow chart of the analysis of the delay time for planned admission. (All cases were investigated from January 2019 to December 2020) [file epih-44-e2022044-suppl2.docx]

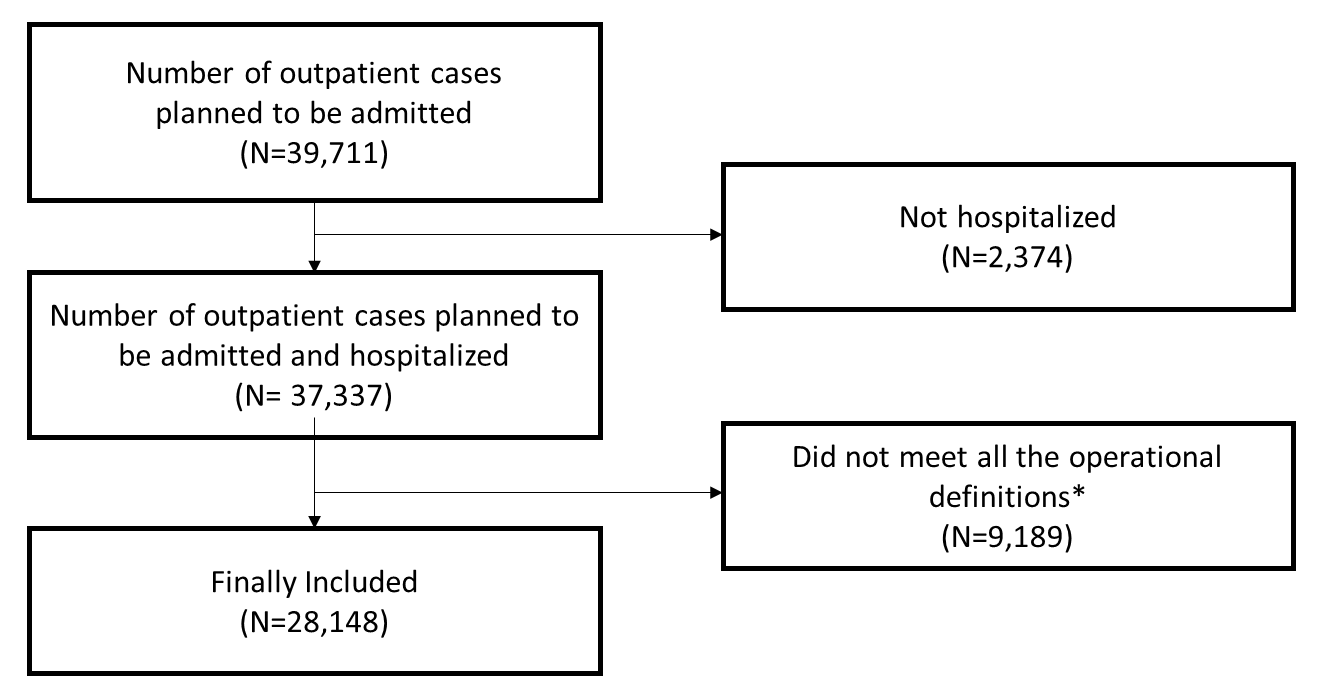


**Supplementary Material 2. Flow chart of the analysis of the delay time for planned admission. (All cases were investigated from January 2019 to December 2020)**

Of 39,711 outpatients eligible for admission, 2,374 were not hospitalized and were thus excluded. Four criteria were used for inclusion in the final dataset as follows: 1) Outpatients who were admitted and hospitalized, 2) outpatients admitted to the same department that referred them, 3) outpatients hospitalized within 3 months, and 4) the nearest admission cases from the date of admission order set were included in the final data. Finally, 9,189 cases who did not meet the operational definitions were excluded and 28,148 were analyzed to determine the average admission delay.

* Operational definitions: (outpatient cases ordered to be admitted and hospitalized) AND (outpatient cases admitted to the same department that referred them) AND (outpatients hospitalized within 3 months) AND (the nearest admission)
