# Supplementary material for: The impact of COVID-19 on cancer care in a tertiary hospital in Korea: possible collateral damage to emergency care
Source: Epidemiol Health. 2022 May 1;44:e2022044. doi: 10.4178/epih.e2022044 (PMC9684015; doi:10.4178/epih.e2022044)
Supplement: Supplementary Material 3. — Percent change of delay time for planned admission according to various factors. (A) Percent change of delay time for planned admission by age group. (B) Percent change of delay time for planned admission by sex. (C) Percent change of delay time for planned admission by type of insurance. [file epih-44-e2022044-suppl3.docx]

**A**


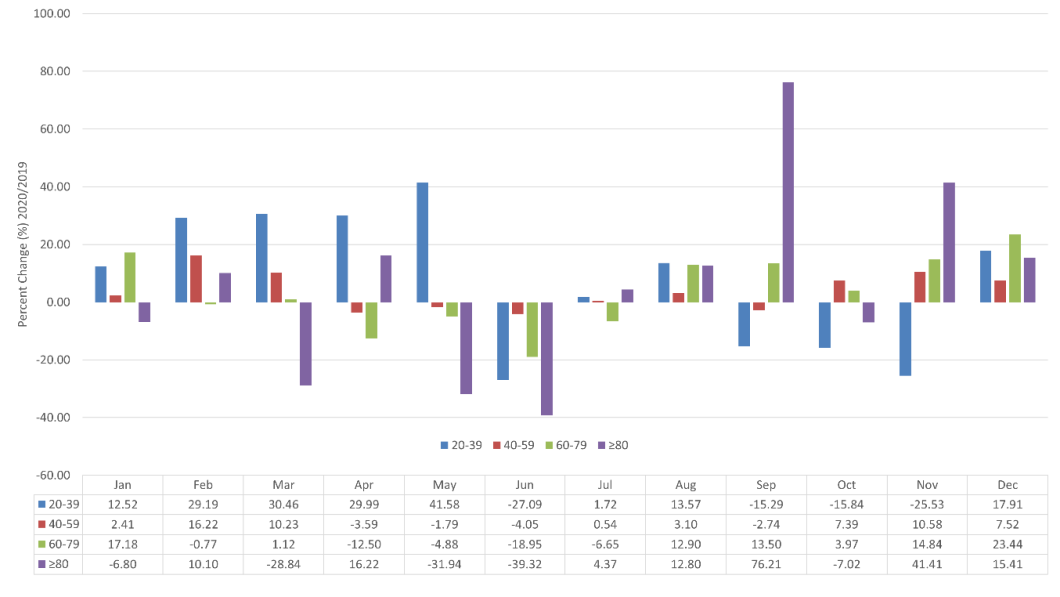


**B**


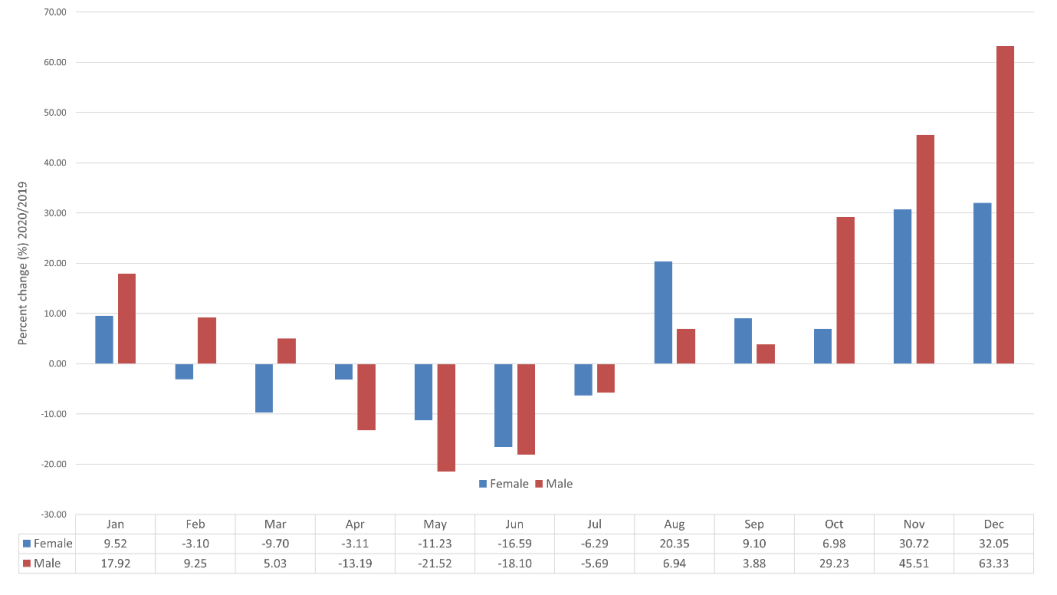


**C**


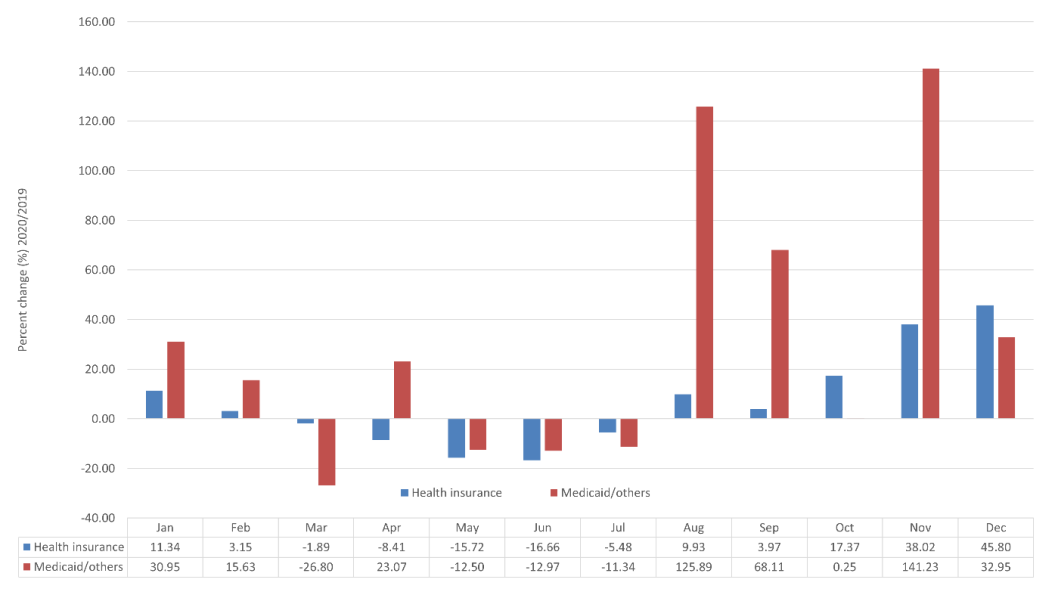


**Supplementary Material 3**. Percent change of delay time for planned admission according to various factors. **(A**) Percent change of delay time for planned admission by age group. **(B**) Percent change of delay time for planned admission by sex. (**C**) Percent change of delay time for planned admission by type of insurance.
