# Supplementary material for: The impact of COVID-19 on cancer care in a tertiary hospital in Korea: possible collateral damage to emergency care
Source: Epidemiol Health. 2022 May 1;44:e2022044. doi: 10.4178/epih.e2022044 (PMC9684015; doi:10.4178/epih.e2022044)
Supplement: Supplementary Material 4. — Percent changes of monthly ED visits stratified by various factors. (A) Percent changes of monthly ED visits by age group. (B) Percent change of monthly ED visits by sex. (C) Percent change of ED visits by residence. (D) Percent change of monthly ED visits by reason for visit. ED, emergency department. [file epih-44-e2022044-suppl4.docx]

**A**


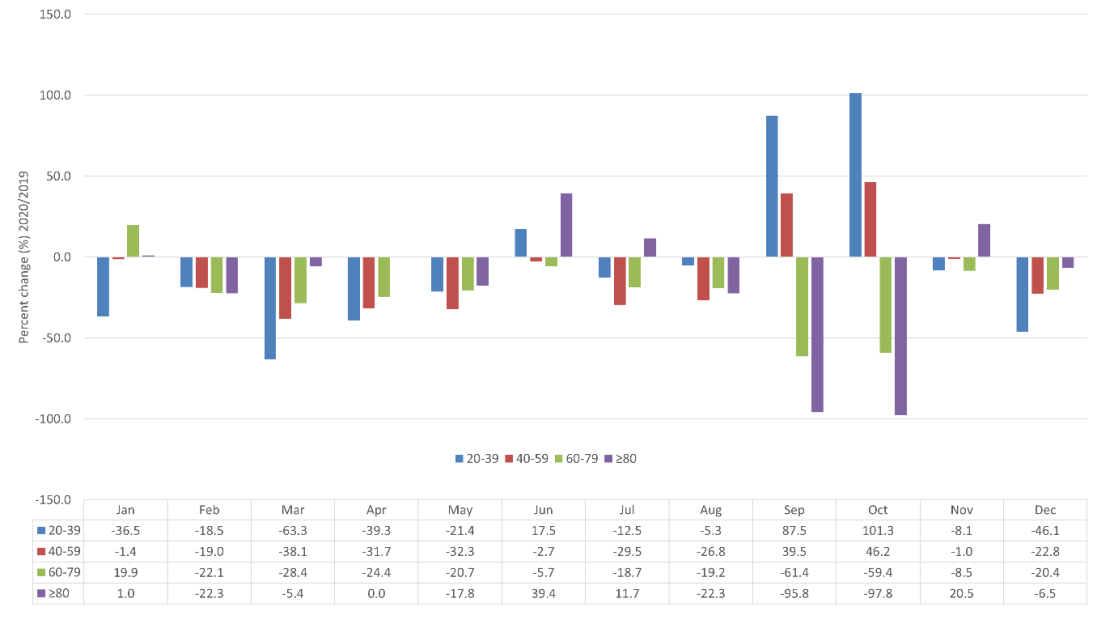


**B**

**
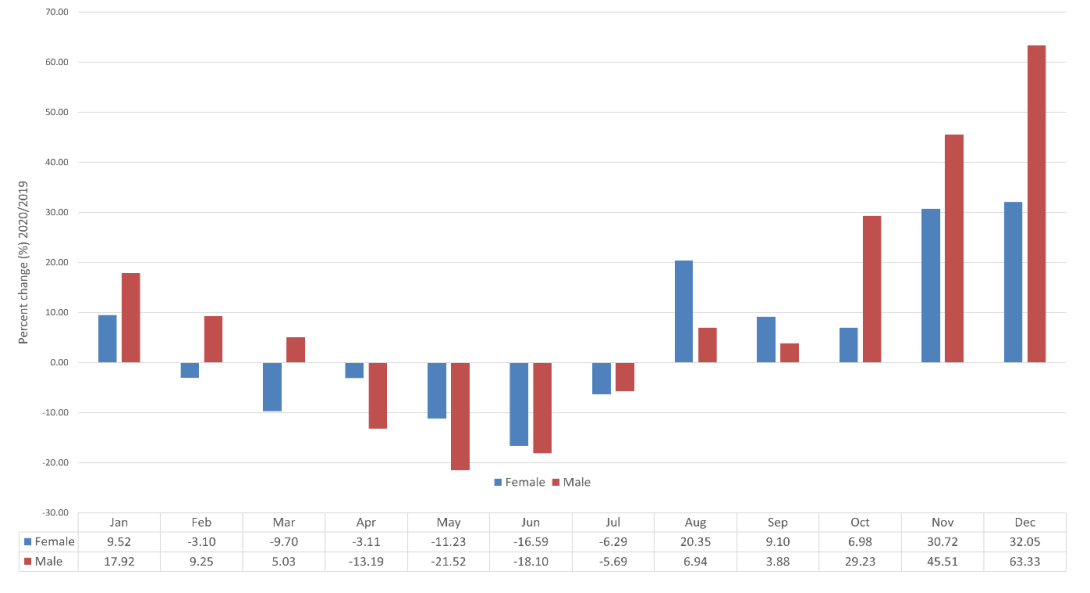
**

**C**


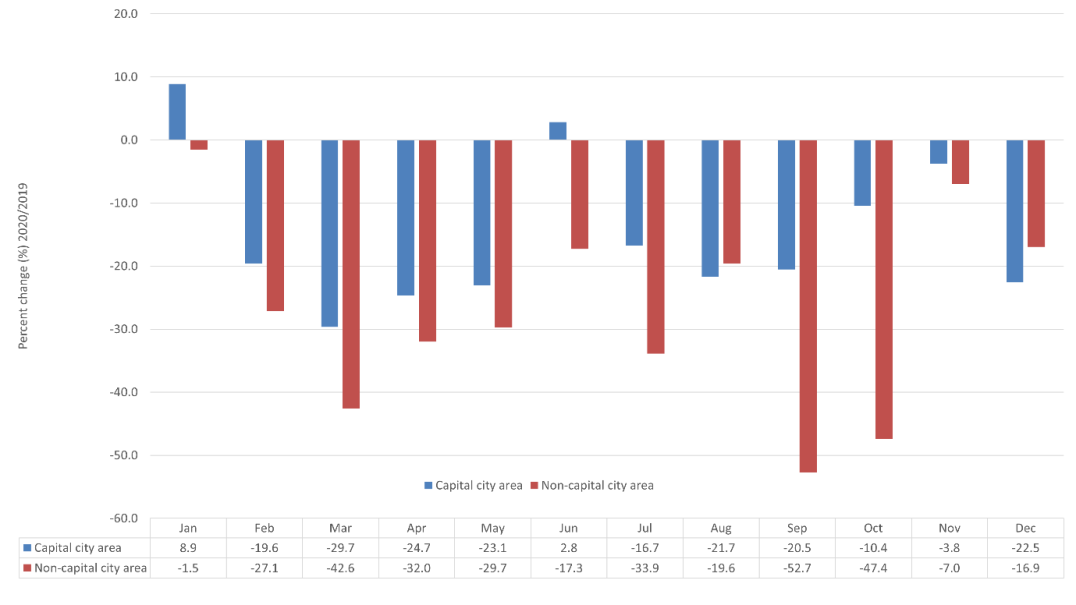


**D**


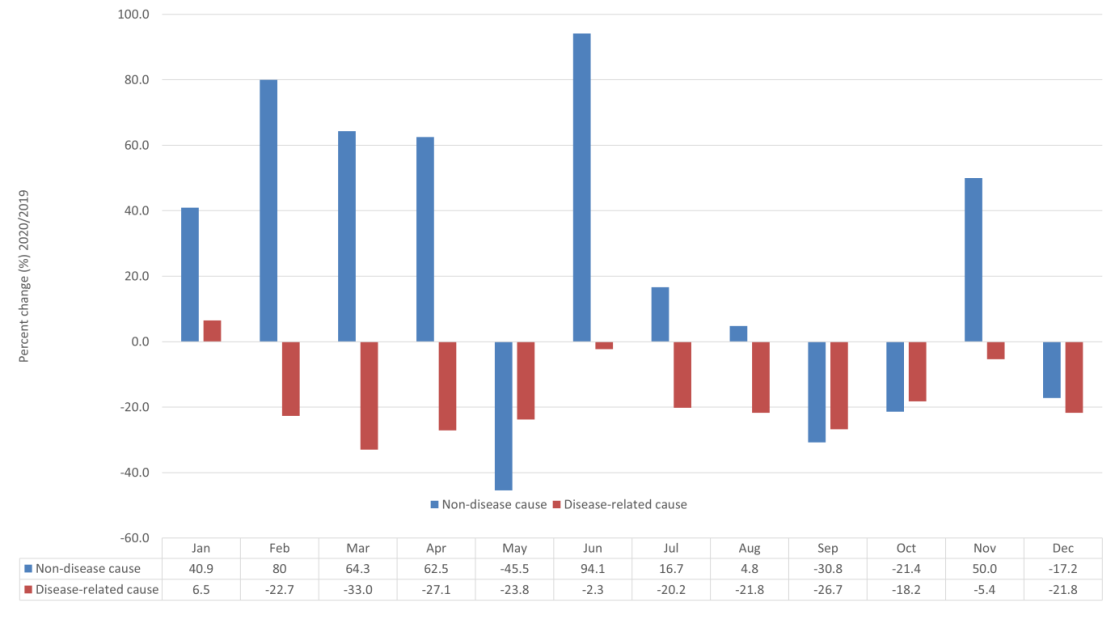


**Supplementary Material 4.** Percent changes of monthly ED visits stratified by various factors. (**A)** Percent changes of monthly ED visits by age group. **(B)** Percent change of monthly ED visits by sex. (**C)** Percent change of ED visits by residence. **(D)** Percent change of monthly ED visits by reason for visit.

ED, emergency department.
