# Supplementary material for: The impact of COVID-19 on cancer care in a tertiary hospital in Korea: possible collateral damage to emergency care
Source: Epidemiol Health. 2022 May 1;44:e2022044. doi: 10.4178/epih.e2022044 (PMC9684015; doi:10.4178/epih.e2022044)
Supplement: Supplementary Material 5. — Number of monthly ED visits by cancer type [file epih-44-e2022044-suppl5.docx]

**Supplementary Material 5. Number of monthly ED visits by cancer type**

| Cancer type^a)^ | Year | Jan | Feb | Mar | Apr | May | Jun | Jul | Aug | Sep | Oct | Nov | Dec |
| --- | --- | --- | --- | --- | --- | --- | --- | --- | --- | --- | --- | --- | --- |
| Lip, oral cavity, and pharynx | 2019 | 13 | 20 | 22 | 18 | 22 | 23 | 18 | 11 | 17 | 15 | 26 | 34 |
|  | 2020 | 26 | 15 | 12 | 11 | 11 | 13 | 21 | 9 | 8 | 17 | 16 | 16 |
| Gastrointestinal | 2019 | 230 | 187 | 238 | 211 | 200 | 156 | 224 | 236 | 206 | 189 | 170 | 180 |
|  | 2020 | 198 | 145 | 141 | 145 | 174 | 174 | 207 | 175 | 139 | 184 | 165 | 150 |
| Hepatobiliary-pancreas | 2019 | 307 | 326 | 388 | 352 | 358 | 279 | 396 | 396 | 343 | 359 | 291 | 295 |
|  | 2020 | 337 | 256 | 270 | 256 | 269 | 288 | 344 | 291 | 297 | 331 | 298 | 264 |
| Lung and intrathoracic | 2019 | 140 | 167 | 143 | 183 | 184 | 162 | 167 | 152 | 156 | 170 | 156 | 154 |
|  | 2020 | 141 | 123 | 109 | 108 | 127 | 157 | 127 | 124 | 104 | 114 | 117 | 122 |
| Bone and soft tissue | 2019 | 24 | 21 | 39 | 22 | 33 | 16 | 30 | 26 | 26 | 28 | 28 | 30 |
|  | 2020 | 24 | 18 | 25 | 27 | 14 | 24 | 26 | 19 | 26 | 19 | 18 | 24 |
| Breast | 2019 | 107 | 111 | 113 | 125 | 135 | 111 | 140 | 118 | 131 | 124 | 91 | 111 |
|  | 2020 | 110 | 94 | 74 | 74 | 75 | 86 | 92 | 75 | 87 | 88 | 76 | 76 |
| Gynecological | 2019 | 63 | 59 | 62 | 59 | 71 | 63 | 98 | 79 | 96 | 67 | 54 | 78 |
|  | 2020 | 63 | 35 | 58 | 71 | 54 | 65 | 60 | 72 | 62 | 55 | 63 | 54 |
| Genitourinary | 2019 | 74 | 93 | 109 | 99 | 122 | 70 | 149 | 113 | 115 | 107 | 104 | 103 |
|  | 2020 | 122 | 79 | 87 | 92 | 93 | 88 | 101 | 93 | 68 | 87 | 121 | 83 |
| Lymphoma | 2019 | 29 | 28 | 28 | 28 | 31 | 26 | 40 | 28 | 25 | 44 | 34 | 32 |
|  | 2020 | 29 | 19 | 18 | 13 | 17 | 31 | 30 | 32 | 27 | 17 | 33 | 13 |
| Leukemia | 2019 | 29 | 28 | 28 | 28 | 31 | 26 | 40 | 28 | 25 | 44 | 34 | 32 |
|  | 2020 | 29 | 19 | 18 | 13 | 17 | 31 | 30 | 32 | 27 | 17 | 33 | 13 |
| Other hematological | 2019 | 50 | 58 | 58 | 48 | 60 | 66 | 51 | 73 | 70 | 54 | 50 | 72 |
|  | 2020 | 73 | 51 | 37 | 47 | 67 | 56 | 55 | 51 | 44 | 36 | 52 | 48 |
| Others | 2019 | 70 | 54 | 83 | 67 | 76 | 50 | 76 | 56 | 73 | 63 | 68 | 58 |
|  | 2020 | 55 | 45 | 40 | 53 | 67 | 55 | 60 | 63 | 42 | 47 | 65 | 43 |

^a)^ Cancer type classification (by ICD-10 codes): lip, oral cavity, and pharynx (C00–C14); gastrointestinal (C15–C21, C26); hepatobiliary-pancreas (C22–C25); lung and intrathoracic (C30–C39); bone and soft tissue (C40–C41, C45–C49); breast (C50); gynecological (C51–C58); genitourinary (C60–C68); lymphoma (C81–C85); leukemia (C91–C95); other hematological (C86, C88, C90, C96, D45–D48); others including malignant neoplasms of the skin (C43–44), central nervous system (C69–C72), endocrine glands (C73–C75), and metastatic or unknown origin (C76–C80).
