# Supplementary material for: The impact of COVID-19 on cancer care in a tertiary hospital in Korea: possible collateral damage to emergency care
Source: Epidemiol Health. 2022 May 1;44:e2022044. doi: 10.4178/epih.e2022044 (PMC9684015; doi:10.4178/epih.e2022044)
Supplement: Supplementary Material 6. — Discharge from emergency department outcomes for the pre-COVID-19 and during-COVID-19 periods and their associations with chief complaints. (A) Monthly discharge outcome from the emergency department. (B) Discharge to home as the outcome stratified by chief complaint. (C) Transfer to other hospital as discharge outcome stratified by chief complaint. (D) Admission as discharge outcome stratified by chief complaint. [file epih-44-e2022044-suppl6.docx]

**A**


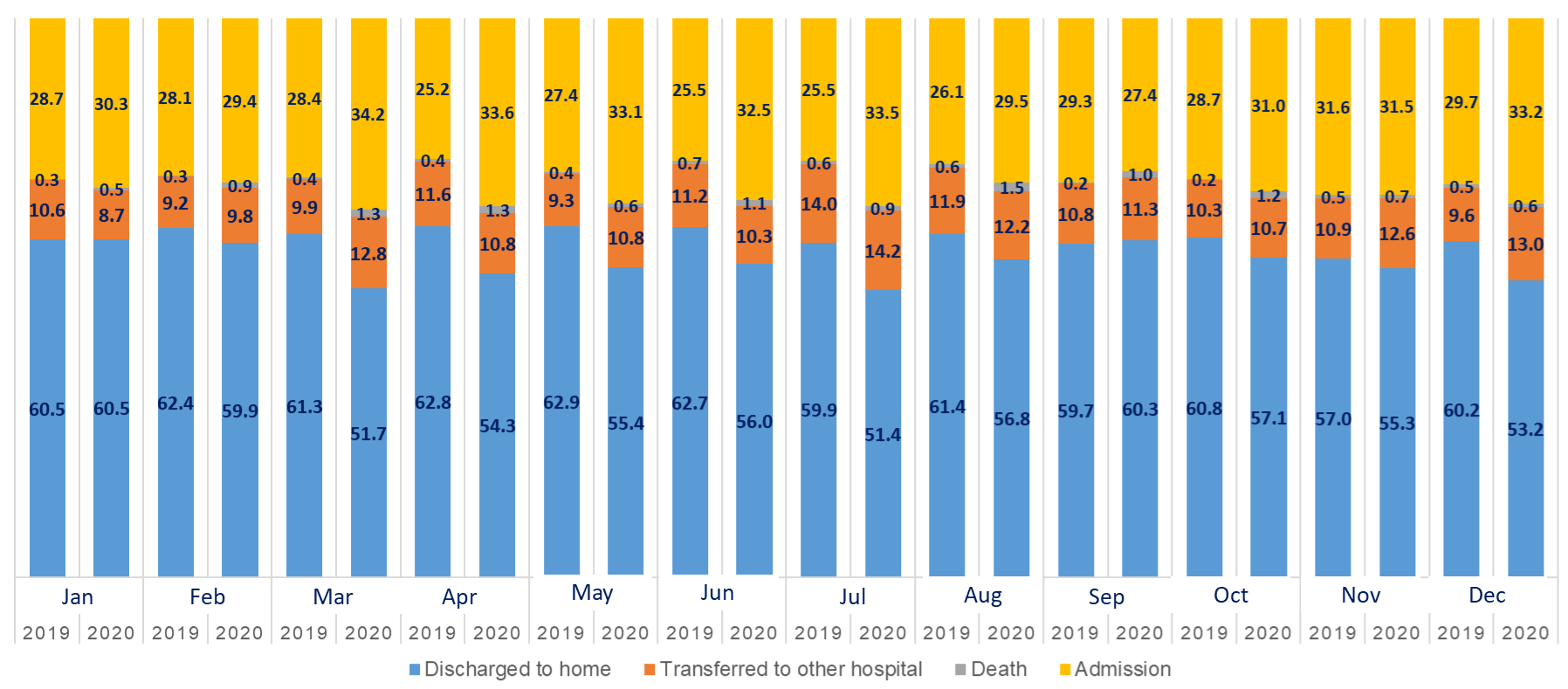


**B**


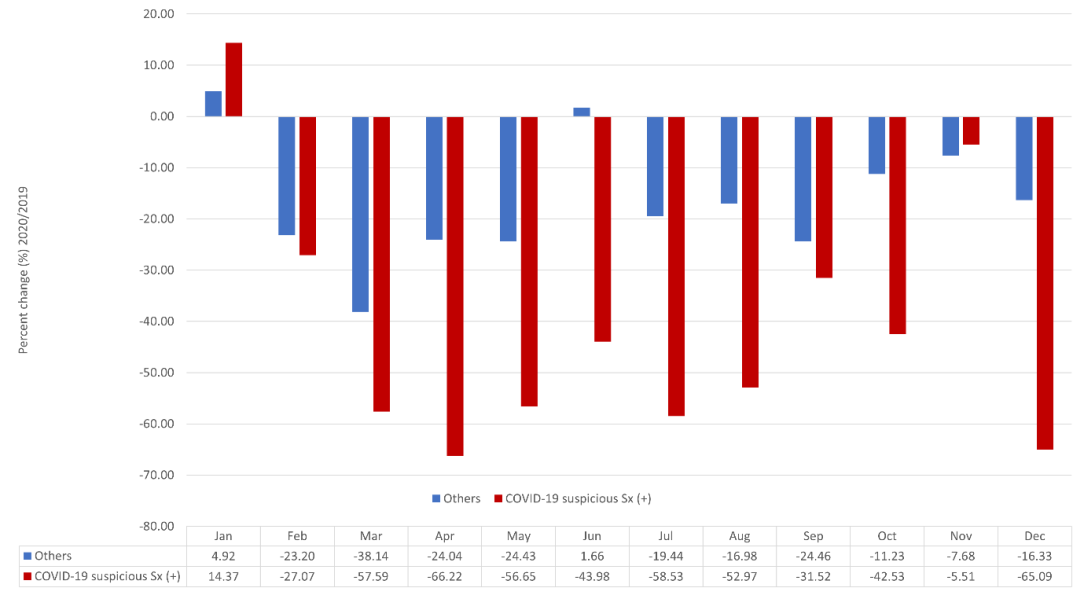


**C**


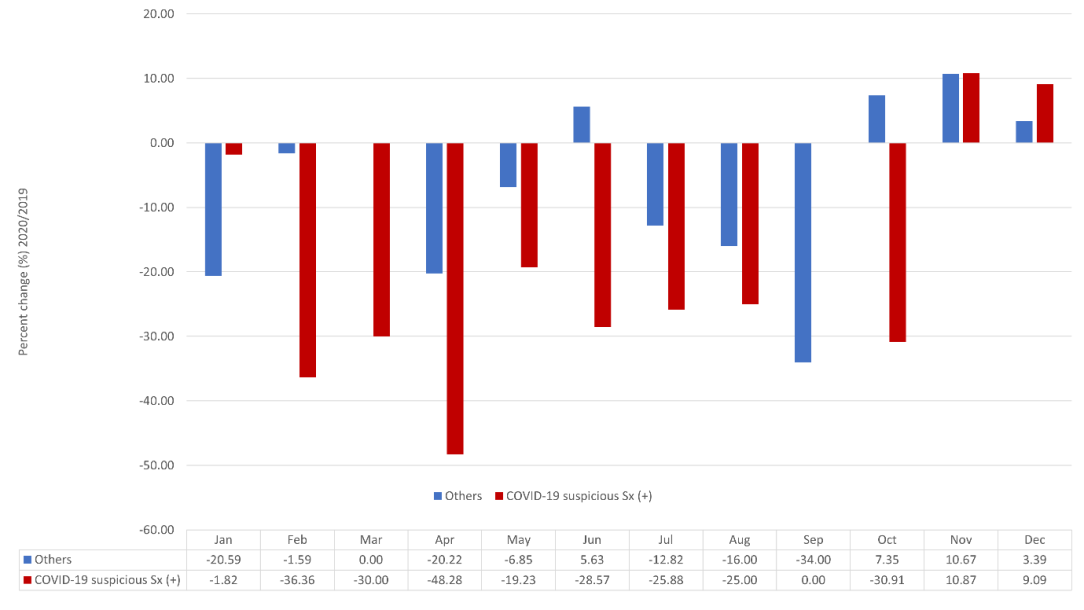


**D**


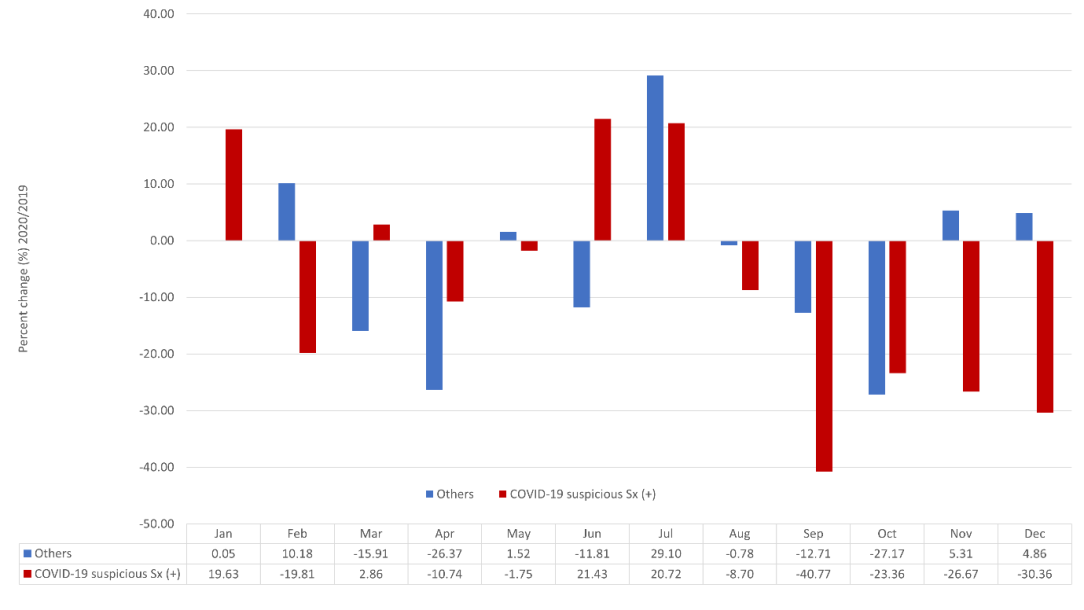


**Supplementary Material 6**. Discharge from emergency department outcomes for the pre-COVID-19 and during-COVID-19 periods and their associations with chief complaints. (**A**) Monthly discharge outcome from the emergency department. (**B**) Discharge to home as the outcome stratified by chief complaint. (**C**) Transfer to other hospital as discharge outcome stratified by chief complaint. (**D**) Admission as discharge outcome stratified by chief complaint.
