# Supplementary material for: The impact of COVID-19 on cancer care in a tertiary hospital in Korea: possible collateral damage to emergency care
Source: Epidemiol Health. 2022 May 1;44:e2022044. doi: 10.4178/epih.e2022044 (PMC9684015; doi:10.4178/epih.e2022044)
Supplement: Supplementary Material 7. — Hospital admissions in the pre-COVID-19 and during-COVID-19 periods. (A) Number of admissions. (B) Average length of hospital stay. (C) Overall rate of admissions via the emergency department. (D) Rate of admissions via the emergency department stratified by residence. [file epih-44-e2022044-suppl7.docx]

| **A** | **B** |
| --- | --- |
| 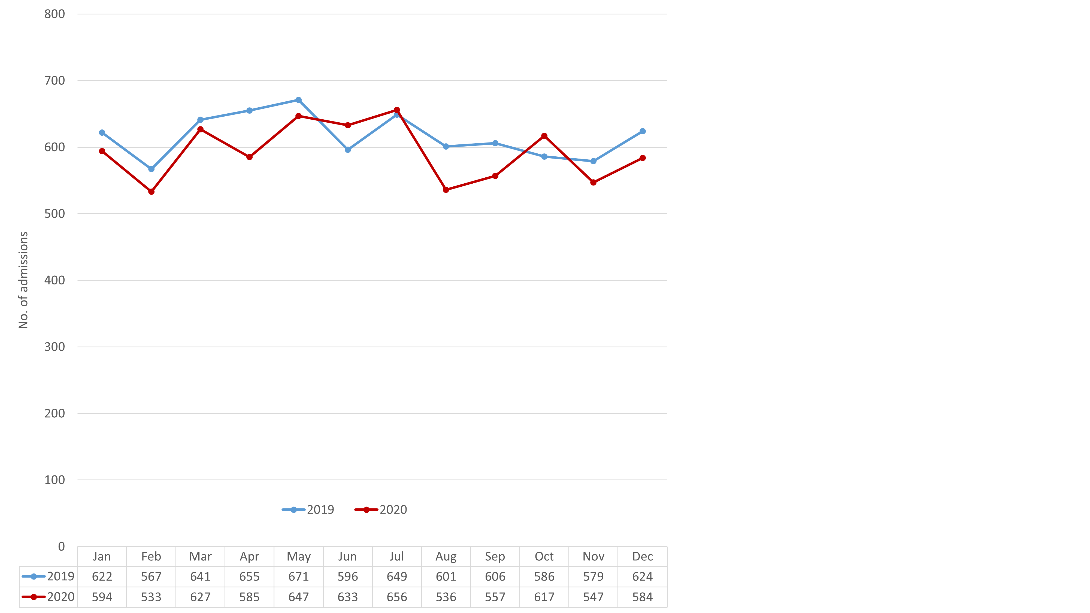 | 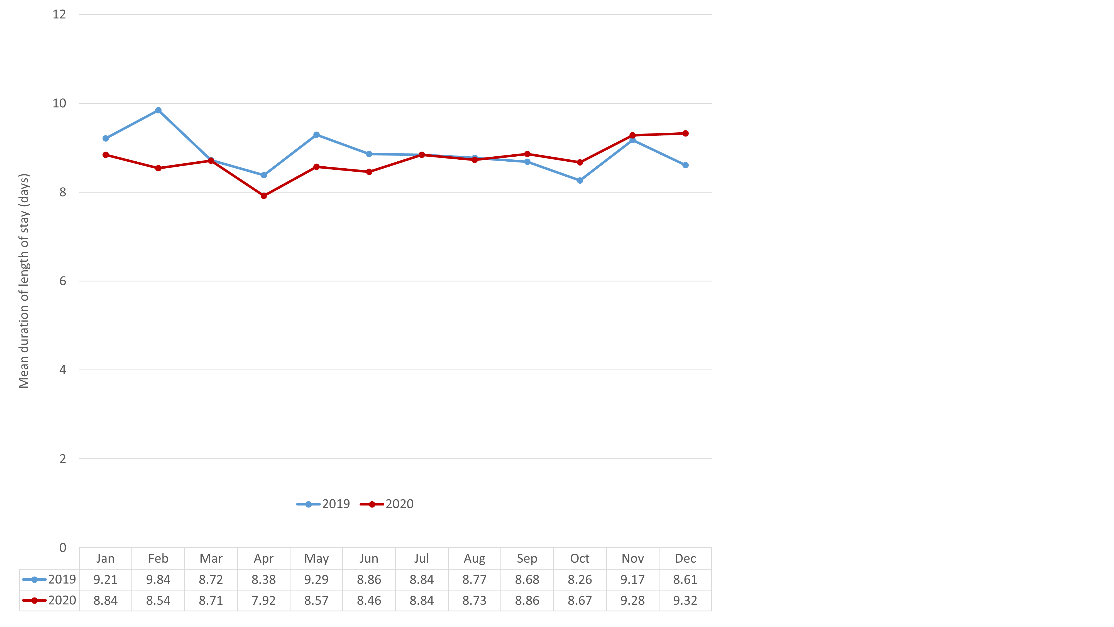 |
| **C** | **D** |
| 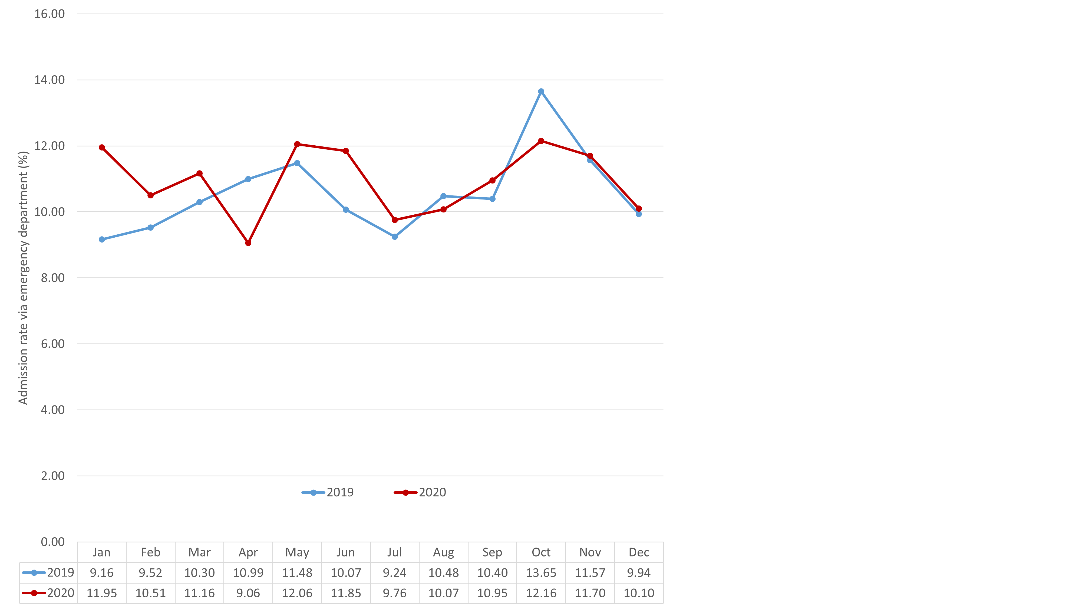 | 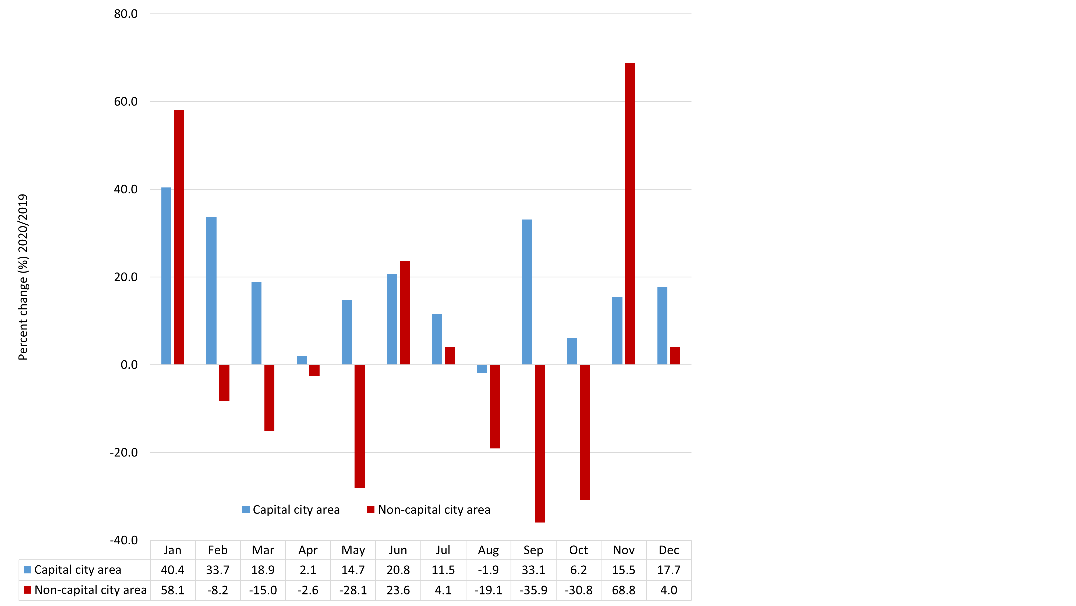 |

**Supplementary Material 7**. Hospital admissions in the pre-COVID-19 and during-COVID-19 periods. (**A**) Number of admissions. (**B**) Average length of hospital stay. (**C**) Overall rate of admissions via the emergency department. (**D**) Rate of admissions via the emergency department stratified by residence.
