# Supplementary material for: The impact of COVID-19 on cancer care in a tertiary hospital in Korea: possible collateral damage to emergency care
Source: Epidemiol Health. 2022 May 1;44:e2022044. doi: 10.4178/epih.e2022044 (PMC9684015; doi:10.4178/epih.e2022044)
Supplement: Supplementary Material 8. — Monthly cases of admission stratified by demographic characteristics [file epih-44-e2022044-suppl8.docx]

| Variables | Description | Year | Jan | Feb | Mar | Apr | May | Jun | Jul | Aug | Sep | Oct | Nov | Dec |
| --- | --- | --- | --- | --- | --- | --- | --- | --- | --- | --- | --- | --- | --- | --- |
| **Sex** | Female | 2019 | 267 | 237 | 260 | 269 | 268 | 240 | 285 | 251 | 280 | 262 | 262 | 269 |
|  |  | 2020 | 257 | 243 | 283 | 250 | 281 | 280 | 282 | 230 | 262 | 295 | 267 | 265 |
|  | Male | 2019 | 355 | 330 | 381 | 386 | 403 | 356 | 364 | 350 | 326 | 324 | 317 | 355 |
|  |  | 2020 | 337 | 290 | 344 | 335 | 366 | 353 | 374 | 306 | 295 | 322 | 280 | 319 |
| **Age group (years)** | 20–30s | 2019 | 54 | 44 | 50 | 53 | 46 | 58 | 63 | 62 | 56 | 69 | 67 | 71 |
|  |  | 2020 | 59 | 52 | 60 | 57 | 73 | 54 | 63 | 61 | 71 | 76 | 62 | 49 |
|  | 40–50s | 2019 | 215 | 192 | 219 | 223 | 228 | 211 | 231 | 208 | 222 | 185 | 196 | 221 |
|  |  | 2020 | 209 | 180 | 226 | 208 | 204 | 214 | 196 | 152 | 186 | 206 | 175 | 170 |
|  | 60–70s | 2019 | 328 | 308 | 347 | 355 | 381 | 309 | 333 | 312 | 310 | 304 | 306 | 317 |
|  |  | 2020 | 314 | 280 | 326 | 310 | 350 | 337 | 374 | 307 | 289 | 319 | 298 | 355 |
|  | ≥80s | 2019 | 25 | 23 | 25 | 24 | 16 | 18 | 22 | 19 | 18 | 28 | 10 | 15 |
|  |  | 2020 | 12 | 21 | 15 | 10 | 20 | 28 | 23 | 16 | 11 | 16 | 12 | 10 |
| **Insurance** | National  Health Insurance | 2019 | 597 | 550 | 612 | 635 | 650 | 571 | 619 | 582 | 581 | 564 | 554 | 609 |
|  |  | 2020 | 577 | 519 | 609 | 570 | 627 | 609 | 635 | 523 | 546 | 596 | 525 | 569 |
|  | Medicaid | 2019 | 23 | 15 | 25 | 17 | 17 | 23 | 26 | 13 | 20 | 18 | 21 | 14 |
|  |  | 2020 | 15 | 14 | 18 | 15 | 20 | 24 | 21 | 13 | 11 | 21 | 22 | 14 |
|  | Others | 2019 | 2 | 2 | 4 | 3 | 4 | 2 | 4 | 6 | 5 | 4 | 4 | 1 |
|  |  | 2020 | 2 | 0 | 0 | 0 | 0 | 0 | 0 | 0 | 0 | 0 | 0 | 1 |
| **Residence** | Capital city area^a)^ | 2019 | 396 | 347 | 402 | 417 | 441 | 385 | 428 | 381 | 383 | 365 | 372 | 397 |
|  |  | 2020 | 373 | 355 | 403 | 376 | 428 | 411 | 411 | 320 | 365 | 399 | 351 | 373 |
|  | Non-capital  city area | 2019 | 226 | 220 | 239 | 238 | 230 | 211 | 221 | 220 | 223 | 221 | 207 | 227 |
|  |  | 2020 | 221 | 178 | 224 | 209 | 219 | 222 | 245 | 216 | 192 | 218 | 196 | 211 |

**Supplementary Material 8. Monthly cases of admission stratified by demographic characteristics**

^a)^ Capital city area includes Seoul-si, Gyeonggi-province, and Incheon-si.
